# Metabolic Regulations by lncRNA, miRNA, and ceRNA Under Grass-Fed and Grain-Fed Regimens in Angus Beef Cattle

**DOI:** 10.3389/fgene.2021.579393

**Published:** 2021-03-04

**Authors:** Cunling Jia, Ying Bai, Jianan Liu, Wentao Cai, Lei Liu, Yanghua He, Jiuzhou Song

**Affiliations:** ^1^College of Animal Science and Technology, Northwest A&F University, Yangling, China; ^2^Department of Animal & Avian Science, University of Maryland, College Park, MD, United States; ^3^Research Centre for Animal Genome, Agricultural Genome Institute at Shenzhen, Chinese Academy of Agricultural Science, Shenzhen, China; ^4^Department of Human Nutrition, Food and Animal Sciences, College of Tropical Agriculture and Human Resources, University of Hawaii, Manoa, HI, United States

**Keywords:** lncRNAs, miRNAs, ceRNAs, beef cattle, feeding regimens, metabolic regulations

## Abstract

Beef cattle raised under grass-fed and grain-fed have many differences, including metabolic efficiency and meat quality. To investigate these two regimens' intrinsic influence on beef cattle, we used high-throughput sequencing and metabolomics analyses to explore differentially expressed genes (DEGs) and metabolimic networks in the liver. A total of 200 DEGs, 76 differentially expressed miRNAs (DEmiRNAs), and two differentially expressed lncRNAs (DElncRNAs) were detected between regimen groups. Metabolic processes and pathways enriched functional genes including target genes of miRNAs and lncRNAs. We found that many genes were involved in energy, retinol and cholesterol metabolism, and bile acid synthesis. Combined with metabolites such as low glucose concentration, high cholesterol concentration, and increased primary bile acid concentration, these genes were mainly responsible for lowering intramuscular fat, low cholesterol, and yellow meat in grass-fed cattle. Additionally, we identified two lncRNAs and eight DEGs as potential competing endogenous RNAs (ceRNAs) to bind miRNAs by the interaction network analysis. These results revealed that the effects of two feeding regimens on beef cattle were mainly induced by gene expression changes in metabolic pathways mediated via lncRNAs, miRNAs, and ceRNAs, and contents of metabolites in the liver. It may provide a clue on feeding regimens inducing the metabolic regulations.

## Introduction

Feeding regimens of beef cattle influence meat quality, growth rate, greenhouse emission, and animal welfare. Many studies reported the characteristic differences of beef cattle between grass-fed and grain-fed including the fat acid compositions, intramuscular fat and cholesterol contents, tenderness and flavor in the muscle (Sithyphone et al., 2011; Orime et al., 2012; Zhao et al., 2012; Li et al., 2015a,b; Carrillo et al., 2016; Berger et al., 2018; Mapato and Wanapat, 2018; Holman et al., 2019; Puzio et al., 2019). For grass-fed beef cattle, the growth rate reaching to market weight is slower than that of grain-fed (Cheung and McMahon, 2017), the intramuscular fat and cholesterol content are also low (Dunne et al., 2009) in beef, but omega-3: omega-6 fatty acids and CLA contents are high (Pavan and Duckett, 2013; Lobato et al., 2014). Meat intake from grass-fed cattle for an adult will significantly increase consumers' long-chain omega-3 PUFA content of plasma and platelet (McAfee et al., 2011), which will reduce the risk of coronary heart disease and resist thrombotic and inflammatory diseases (Mozaffarian et al., 2005). Besides, the grass-fed regimen can also improve animal welfare, eliminate risks of bovine spongiform encephalopathy (Lobato et al., 2014), and decrease carbon footprints (Lynch, 2019).

What is the molecular mechanism of inducing these differences between the two regimens? We had previously analyzed the possible mechanism based on transcriptome and metabolomics in the rumen (Li et al., 2015b), spleen (Li et al., 2015a), and muscle (Carrillo et al., 2016). Many identified differentially expressed genes (DEGs) were associated with a lower total fat and a higher omega-3/omega-6 ratio (Carrillo et al., 2016) in grass-fed cattle. Moreover, some other DEGs were associated with substance transport, organ and organism development in the rumen (Li et al., 2015b), with increasing immunity in the spleen (Li et al., 2015a), and with less stress for grass-fed cattle (Carrillo et al., 2016). Under two feeding regimens, the diet structure is different. A grass-fed diet has lower non-fibrous carbohydrates (NFC) and higher Neutral Detergent Fiber (NDF) than a grain-fed diet. Besides, the rearing environment and management patterns are also different. All these may induce metabolic differences in organisms and organs. As a result, there are many other characteristics for beef cattle under two regimens.

As an essential metabolic organ, the liver can detoxify various metabolites and produce biochemicals necessary for digestion. It also involves many functions such as bile production and excretion, cholesterol metabolism, hormones excretion, metabolism of fats, proteins, and carbohydrates (Mitra and Metcalf, 2009). At present, it is unclear how two feeding regimens affect the biological processes in the liver.

The changes in nutrition or/and environment can modify gene expression, involving epigenetic regulation. Although its precise role is difficult to be established because of multiple interactions between dietary components and other epigenetic regulators (Dauncey, 2012; Jiménez-Chillarón et al., 2012), it is worthy to mine the relationship between dietary regulating genes and epigenetic regulators.

So far, we have known different non-coding RNAs (ncRNAs) are involved in epigenetics processes. For example, long non-coding RNA (lncRNA) is an ncRNA class with more than 200 nucleotides in length. In mammalian genomes, plenty of lncRNAs are engaged in different biological processes through diverse mechanisms, including the function as scaffolds, decoys or signals, regulation of gene expression in *cis*, or *trans* antisense interference (Kung et al., 2013). MicroRNA (miRNA) is also a type of ncRNAs with 18–25 nucleotides in length. It binds to the complementary sequence mainly in the 3'UTR of target mRNA and thereby regulates gene expression (Bartel, 2004; Winter et al., 2009). Previous studies reported that the expression of miRNAs and lncRNAs correlated with diet and lifestyle (Palmer et al., 2014; Slattery et al., 2017; Silva and van Booven, 2018). LncRNAs can act as molecular sponges of miRNAs through microRNA response elements (MREs) and function as a competing endogenous RNA (ceRNA) to protect mRNA (Ebert et al., 2007; Tay et al., 2014). The coding and noncoding RNA can interact with each other through MREs and form large-scale regulatory networks across the transcriptome, which will make sense to uncover the mechanism of biology phenomenon (Salmena et al., 2011).

In this study, we hypothesize that different metabolisms exist in the liver associated with beef cattle's characteristic differences under two feeding regimens. To test it, we performed transcriptome analyses in the liver and metabolomics analyses in blood to identify functional genes, pathways, and metabolites and construct a regulatory network. We believe these results would provide a deep insight into the metabolic mechanisms and benefit the beef industry to produce healthier and higher quality beef.

## Materials and Methods

### Ethics Approval

All experiments were conducted using procedures approved by the Institute of Animal Care and Use Committee at the University of Maryland.

### Animals and Samples Collection

Liver samples of Wye Angus steer under grass-fed and grain-fed were collected when they reached market weight (the average final weights and ages were 459.6 ± 35.87 kg, 14 months old for the grain-fed steers and 471.1 ± 36.49 kg, 21 months old for grass-fed steers). Before slaughtering, 10 ml of whole blood from the jugular vein was collected in EDTA tubes and directly stored at −80°C for metabolite measurement. After killing, six individuals' liver samples located at lobus hepatis sinister from the grass-fed and grain-fed group (each group of three individuals) were collected, immediately frozen in dry ice, returned to the laboratory, and frozen at −80°C for farther analyses. Since this population has been closed for more than 70 years, all individuals have a similar genetic background. The diet composes of grass-fed (mainly including alfalfa, hay, or grass pasture) or grain-fed (mainly including corn silage, shelled corn, soybean, and minerals, etc.), and feeding regimens were the same as the description from Carrillo et al. (2016). The diet nutrition components of grass-fed and grain-fed cattle were different: soluble protein 28 and 47%, respectively, the ratio of non-fiber carbohydrates and neutral detergent fiber (NFC: NDF) 0.33 and 1.59, starch 0.2 and 35.6%, total digestible nutrients 60 and 73%, net energy (for milk, maintain, and gain) 1.42 and 2.07 Mcal/lb based on the dry mess (Carrillo et al., 2016).

### Library Preparation and High-Throughput Sequencing for mRNA and miRNA

According to the manual instruction, total RNA was extracted and purified from liver samples using the RNAeasy® Plus Mini Kit (Qiagen, Valencia, CA). The concentration of RNA was accessed by a Nanodrop ND-2000 spectrophotometer (Thermo Fisher Scientific, DE, USA). The RNA integrity (RIN) was checked by the Bioanalyzer 2100 system (Agilent Technologies, CA, USA), and RIN was more than 7.0. The cDNA libraries were built using the NEBNext® Ultra™ RNA Library Prep Kit for Illumina® (NEB, USA). The Agilent Bioanalyzer 2100 system was used to measure the libraries' quality for RNA-seq from each sample of grass-fed cattle and grain-fed cattle. Each library was sequenced in 50 bp reads length using the Illumina® HiSeq 2000 platform (Williams et al., 2014; Hrdlickova et al., 2017).

Small RNA with 18–30 nt was obtained from the total RNA, and adapter ligation and RT-PCR were carried out to construct small RNA libraries for six liver samples of grass-fed and grain-fed cattle using TruseqTM Small RNA sample prep kit according to the protocols (Lagos-Quintana et al., 2001). These libraries were sequenced with 50 bp single-end reads on an Illumina HiSeq 2000 platform.

### Reads Quality Control, Alignment, and Annotation

Raw reads were processed by removing adapters and low-quality reads using FastQC (Version 0.11.5) (Andrews and Fast, 2010) to perform quality control. For RNA-seq, reads after filtered and trimmed by Trimmomatic-0.36 (Bolger et al., 2014) were mapped to *Bos taurus* reference genome (ARS-UCD1.2) using Hisat2-2.1.0 (Kim et al., 2019). Small RNA reads with low quality and length <17 or >25 after deleting adapters were removed. Reads were mapped to *Bos taurus* reference genome (ARS-UCD1.2). The known miRNAs were identified based on the miRBase 22.0 (http://www.mirbase.org/) database using miRDeep2 software (Friedländer et al., 2012).

### Identification of DEGs and DEmiRNAs, and Prediction of DEmiRNAs Targets

DEGs were analyzed using cuffdiff (Trapnell et al., 2012), and DEmiRNAs were identified by the EdgeR package in R software (Robinson et al., 2010). Genes with a false discovery rate (FDR) <0.1 were identified as DEGs and DEmiRNAs. We used TargetScan version7.2 (Agarwal et al., 2015) and miRanda (v3.3a) (score cutoff ≥ 140, energy cutoff ≤ -15 kcal/mol, scaling: 4) (Enright et al., 2003) to predict conserved miRNA target sites on the mRNAs. For further analysis, we used common miRNA-targets from both software.

### Mining lncRNA From RNA-seq Data

Based on the Bos taurus reference genome (ARS-UCD1.2) annotated 9,626 lncRNAs (Refseq), we used cuffdiff to calculate fragments per kilobase of exon model per million mapped fragments values and identified possibly DElncRNAs in a grass-fed group vs. grain-fed group from RNA-seq data. To explore the function of lncRNAs, we predicted the target genes of lncRNAs in *cis*- and *trans*-regulation. The *cis*-regulation targets of lncRNAs were searched within 100 kb down-stream and up-stream of DElncRNAs. The potential targets of lncRNA in *trans*-regulation were predicted by calculating the correlation coefficients between lncRNAs and mRNAs. When Spearman correlation coefficients were more than 0.9, DElncRNA-mRNA pairs were regarded as candidate coexpression gene pairs.

### Bioinformatics Analysis of DEGs, Targets of DEmiRNAs and Coexpression Genes of DElncRNAs

We used the online STRING tools (http://string-db.org/) for the Gene Ontology enrichment and KEGG pathways analysis of DEGs, targets of DEmiRNAs, and coexpression genes of DElncRNAs. All enrichment results with FDR <0.05 were deemed to be significant.

### Construction Interaction Network of DElncRNAs, DEmiRNAs, and DEGs

The conserved MREs were predicted in DElncRNAs using miRanda (v3.3a) (Enright et al., 2003). Based on the obtained DEmiRNAs-DEGs, DElncRNAs-DEGs, and DElncRNAs-DEmiRNAs pairs, we constructed an interaction network. The regulatory network was visualized by using the Cytoscape 3.5.0 (http://www.cytoscape.org/).

### Validation of DEGs, DEmiRNAs, and DElncRNAs Expression by Real-Time PCR

We randomly selected six DEGs, six DEmiRNAs, and all DElncRNAs to validate transcriptome sequence reliability using reverse transcription real-time PCR (RT-qPCR). The RT-qPCR primers were designed using Primer Premier 5.0 (http://downloads.fyxm.net/Primer-Premier-101178.html) for DEGs and DElncRNAs. For DEmiRNAs, stem-loop primers were designed for RT-qPCR analysis. All primers were synthesized by Integrated DNA Technologies, Inc., USA. Total RNA of each sample was extracted using the RNAeasy® Plus Mini Kit (Qiagen, Valencia, CA), and 1 μg total RNA was reversely transcribed to cDNA using the QuantiTect® Reverse Transcription Kit (Qiagen, Valencia, CA) for DEGs and DElncRNAs, and using the Taqman MicroRNA Reverse Transcription kit and specific stem-loop RT primers for DEmiRNAs according to manufacturer's instructions. The RT-qPCR was performed using BIORAD iQ™ SYBR® Green Supermix (BIO-RAD, USA) on the BIORAD iQ5 Real-time PCR Detection System. The 10 μl PCR reaction volume included 100 ng RT product, 5 μl 2 × iQ™ SYBR Green supermix, 300 nM forward primers, and 300 nM reverse primer (for all miRNA, using universal reverse primer), and the rest was RNase-free water. We chose *GAPDH* for mRNA and lncRNA and U6 for miRNA as the endogenous control genes. We performed three technical replicates for each sample, and included negative controls without a template. Fold-changes of mRNA, miRNA, and lncRNA expression were calculated using the 2^−Δ*ΔCT*^ method (Livak and Schmittgen, 2001).

### Metabolomics Measure and Analysis

Whole blood samples from 16 individuals (8 samples for each group) were submitted to Metabolon Inc. (Durham, NC, USA) for metabolomic analysis. The extracted samples using Metabolon's standard solvent extraction method were split into equal parts for analysis on the GC/MS and UPLC/MS/MS platforms (Kennedy et al., 2013). Automated comparisons detected the samples' biochemical molecules to the Metabolon's reference library (326 compounds of known identity), and MS/MS patterns of thousands of commercially available purified standard biochemicals tested using the Metabolon's mass spectrometry platform. The combination of chromatographic properties and mass spectra indicated a match to a specific metabolite. The biochemical component's measured method in samples for GC/MS and UPLC/MS/MS was same as described before (Carrillo et al., 2016).

### Statistical Analysis

In metabolomics analysis, following median scaling, imputation of missing values (if any) with the minimum observed value for each compound, and log transformation median scaled data, Welch's two-sample *t*-test was used to identify biochemicals that differed significantly between experimental groups. A statistical significance criterion was set at *P* < 0.05. The *q*-value was estimated to take into account the multiple comparisons. Statistical analyses were performed with the R program (http://cran.r-project.org/).

## Results

### Expression Profile of mRNAs in the Liver From Grass-Fed and Grain-Fed Cattle

To characterize the differences of beef cattle under two regimens, the transcriptomes of the liver were analyzed. A total of 17,900,957 and 20,929,124 clean reads were left for grass-fed and grain-fed groups, respectively. An average of 90% clean reads was mapped to the *Bos taurus* reference genome (Supplementary Table 1). Based on *FDR*'s criterion below 0.1, a total of 200 DEGs were found. Among these, 100 genes were up-regulated and 100 genes were down-regulated in a grass-fed group compared with a grain-fed group (Supplementary Table 2).

### Functional Analysis of DEGs

**Figure 1:**
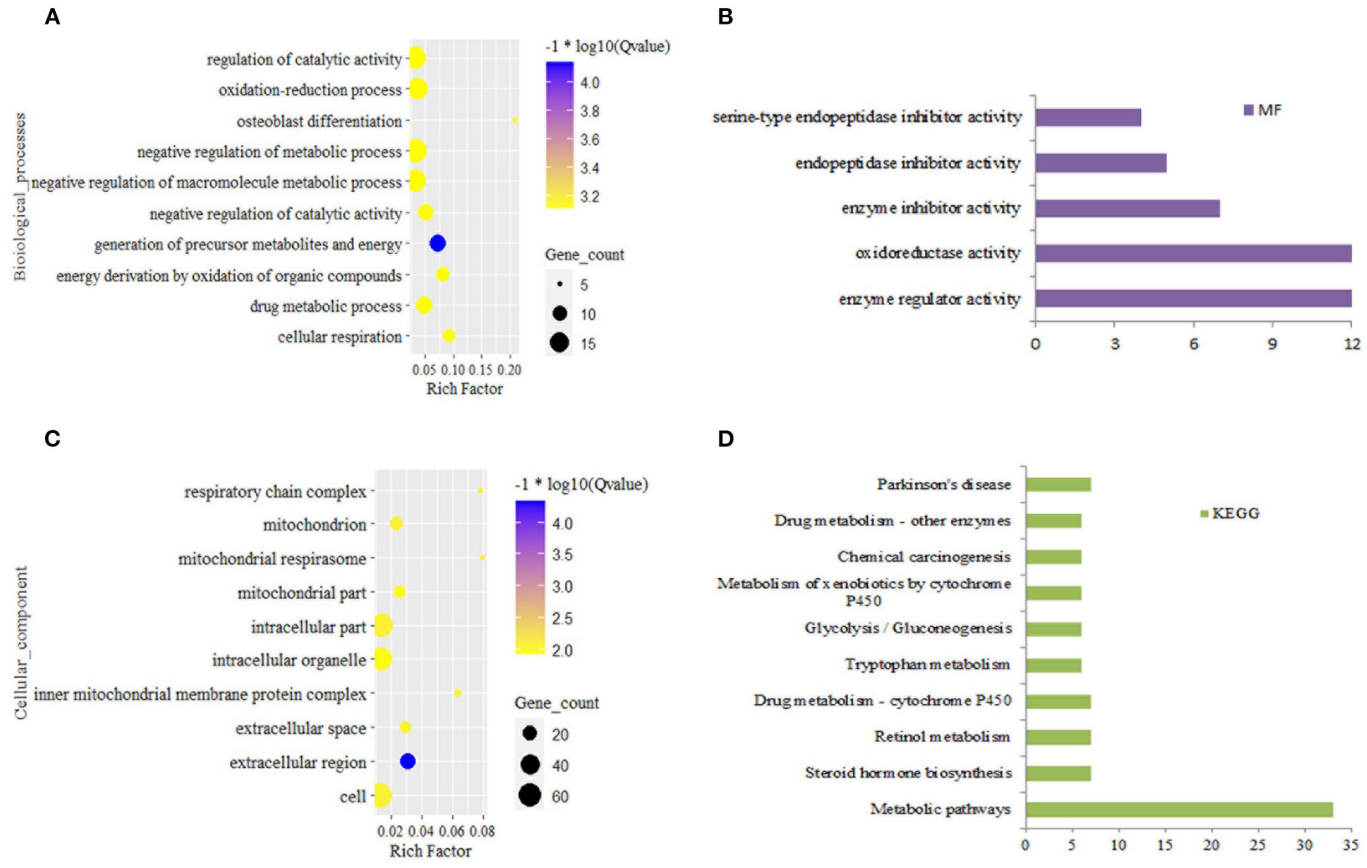
Top 10 significantly enriched function for differential expression gene in grass-fed vs. grain-fed. Biological process **(A)**, molecular function **(B)**, cellular component **(C)**, and KEGG pathways **(D)**.

In the liver, DEGs from grass-fed vs. grain-fed group were enriched to 150 biological processes (BPs), 24 cellular components (CCs), five molecular functions (MFs), 11 KEGG pathways (*FDR* < 0.05) (Supplementary Table 3). Significant GO terms and KEGG pathways were mainly involved in negative regulation of the metabolic process, regulation of catalytic activity, oxidation-reduction process, and metabolic pathway (Figure 1).

### Global miRNA Expression Pattern in the Liver From Grass-Fed and Grain-Fed Cattle

**Figure 2:**
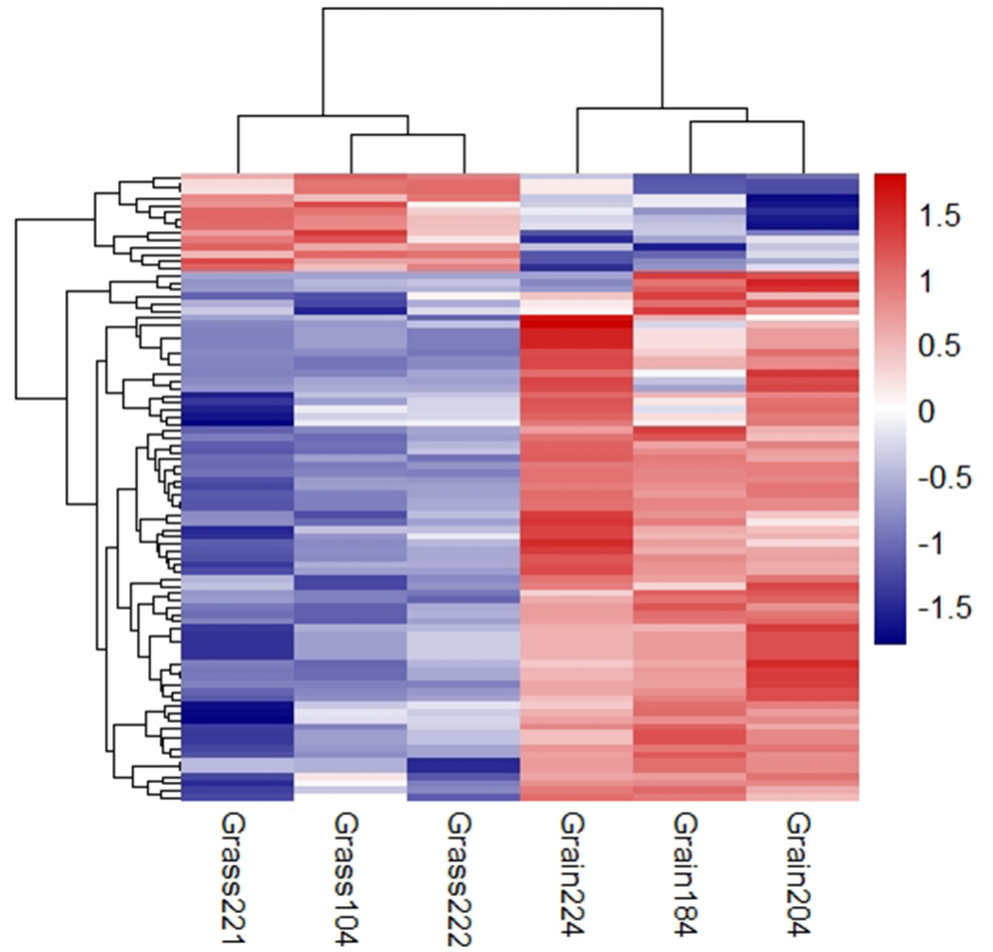
Cluster analysis of differential expression miRNAs in grass-fed vs. grain-fed. On the top-right of the figure, the color difference represents the relative abundance.

The small RNA libraries were constructed from six individual liver samples collected from grass-fed and grain-fed cattle. In total, 54.94 and 54.55 million raw reads were obtained from grass-fed and grain-fed groups, respectively. After filtering the low-quality sequences, 44.38 and 37.42 million clean reads in grass-fed and grain-fed groups were used for further analysis. For grass-fed and grain-fed groups, 57.7 and 48.37% of the cleaned reads were successfully mapped. Known miRNAs were identified based on miRBase 22.0 (http://www.mirbase.org/) using the miRDeep2 software (Friedländer et al., 2012). A total of 445 known mature miRNAs (with count >2 at least two individuals) were detected. After the difference of miRNA expression between grass-fed and grain-fed cattle were analyzed, a total of 76 known mature DEmiRNAs (*FDR* < 0.1) were found. Among these, 64 down-regulated miRNAs and 12 up-regulated miRNAs were detected in grass-fed vs. grain-fed group (Figure 2, Supplementary Table 4).

### Functional Annotation of DEmiRNAs Targets

**Figure 3:**
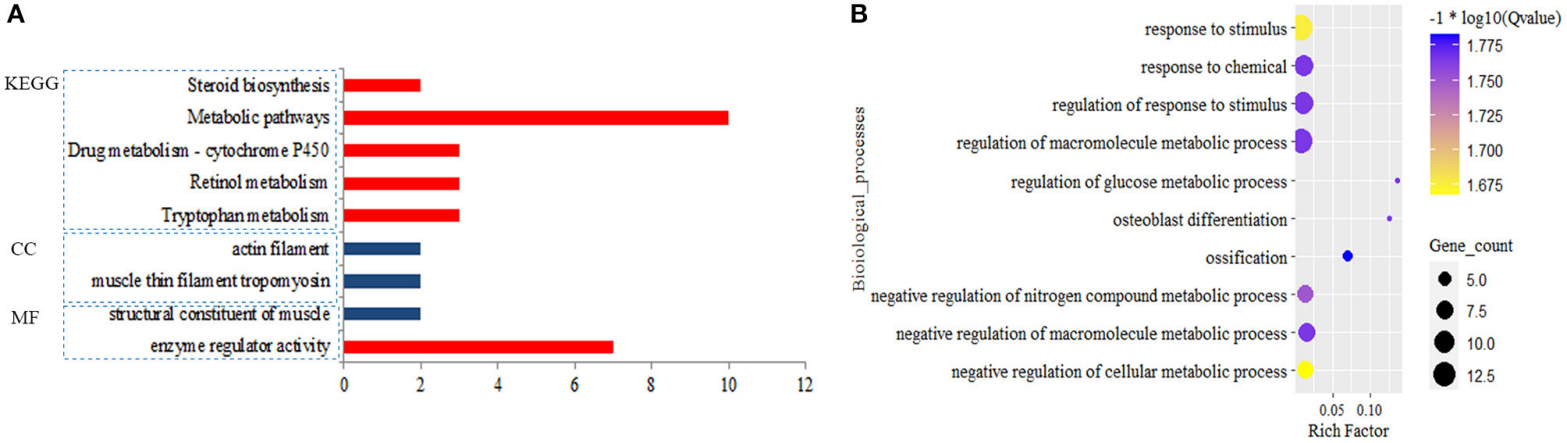
Significantly enriched function for the target genes of differential expression miRNAs (DEmiRNAs). Blue pillar represented the enrichment from down-regulated target genes and red pillar from up-regulated target genes by DEmiRNAs **(A)**; Biological process **(B)**.

A total of 374 DEmiRNAs-DEGs pairs with the reverse relationship were obtained. Functional analysis showed target DEGs of down-regulated DEmiRNAs were enriched to 64 BPs, one MF, and five KEGG pathways. Still, target DEGs of up-regulated miRNAs were only enriched to one MF, two CCs, and no BP and KEGG pathway (*FDR* < 0.05) (Figure 3; Supplementary Table 5). We found that the target DEGs were mainly enriched to the regulation of macromolecule metabolic process,response to stimulus and metabolic pathways.

### Identification and Functional Analysis of Differential Expressed lncRNAs

Based on annotated *Bos taurus* reference genome, we identified two differentially expressed lncRNAs (DElncRNAs) i.e., lnc_ENSBTAT00000076705 and lnc_ENSBTAT00000068696 in liver from RNA-seq data. They were up-regulated in the grass-fed group compared with the grain-fed group. The lnc_ENSBTAT00000076705 was co-located with eight genes (*PTGDR2, MS4A10, CCDC86, TMEM109, TMEM132A, SLC15A3, PRPF19, CD6*), and lnc_ENSBTAT00000068696 was co-located only with one gene (*AGPS*) within a 100 kb window up-stream or down-stream of DElncRNAs through *cis* analysis. Still, all these co-located genes were no significant difference between grass-fed group and grain-fed group. We also performed a coexpression analysis by calculating the expression correlation coefficients between lncRNAs and mRNAs. A total of 141 DElncRNA-mRNA pairs were detected (|r| > 0.9) (Supplementary Table 6). The potiential regulated DEGs enriched to 192 BPs, 9 MFs, 34 CCs, and 15 KEGG pathways (*FD*R < 0.05) (Supplementary Figure 1; Supplementary Table 7). These coexpression DEGs were also mainly enriched to metabolic processes and pathways.

### Construction of DElncRNAs, DEmiRNAs, and DEGs Interaction Networks in Metabolism

**Figure 4:**
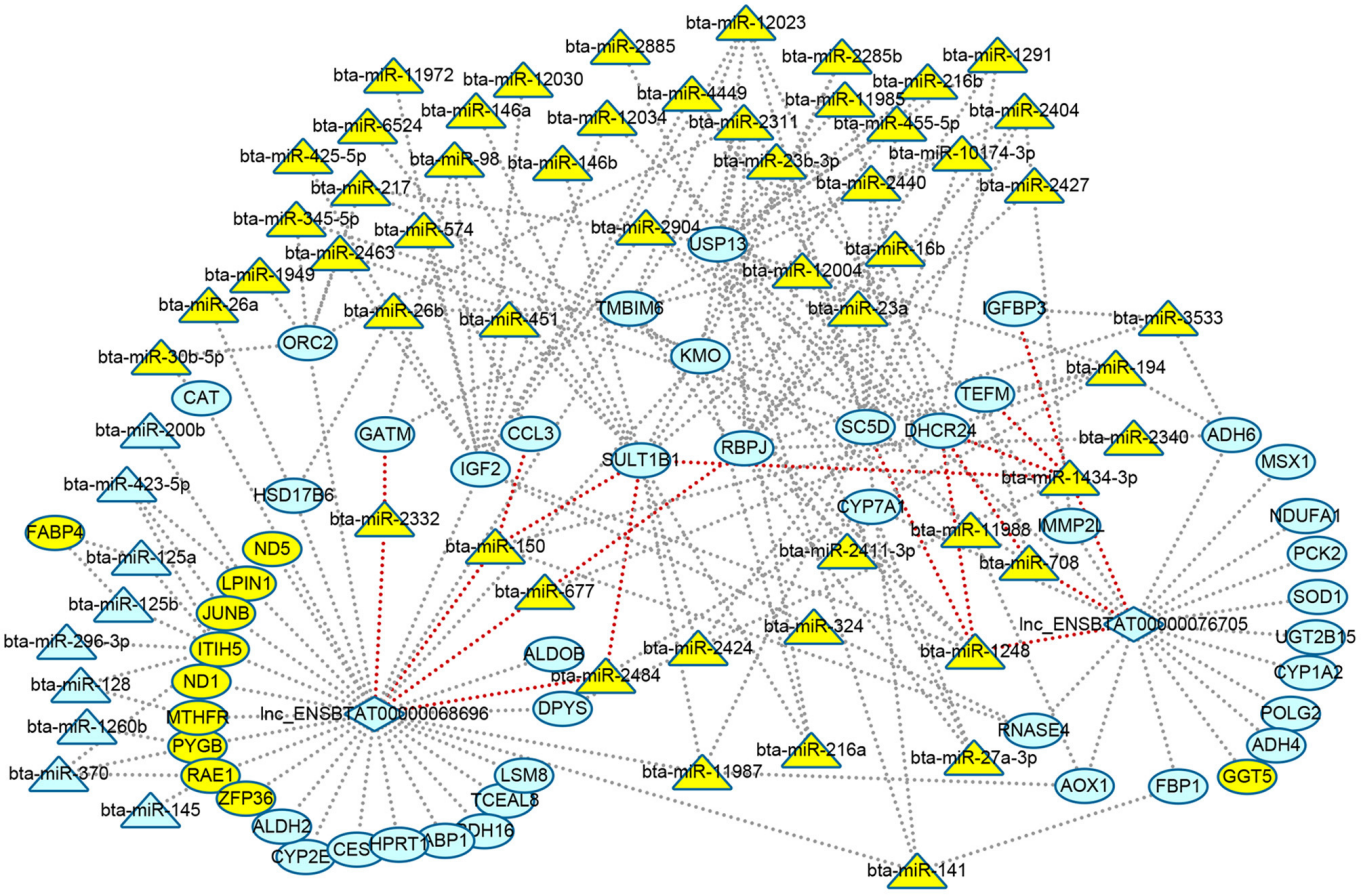
Visualizing regulatory networks of metabolic processes and pathways in liver for grass-fed vs. grain-fed group. Blue represented RNAs up-regulated; green represented RNAs down-regulated; triangle represented miRNAs; circle represented differential expression genes; diamond represented lncRNAs; and red lines represented the edge of lncRNA-miRNA-mRNA network.

The relationship between DElncRNA and DEmiRNA was predicted by miRanda software. As a result, two lncRNAs were related to 11 miRNAs (Supplementary Table 8). Based on the above results of function enrichments, the metabolic processes and pathways were the focus. In order to clarify the metabolic regulating relationship, we constructed an interaction network from DEGs, DElncRNAs, and DEmiRNAs with 114 nodes and 193 edges using Cytoscape (http://www.cytoscape.org/) (Figure 4). We found two lncRNAs, eight DEGs including 24-dehydrocholesterol reductase (*DHCR24*), sterol-C5-desaturase (*SC5D*), glycine amidinotransferase (*GATM)*, sulfotransferase family 1B member 1 (*SULT1B1*), C-C motif chemokine ligand 3 (*CCL3*), recombination signal binding protein for Iimmunoglobulin kappa J region (*RBPJ*), *IGFBP3*, mitochondrial transcription elongation factor (*TEFM*), and seven DEmiRNAs (bta-miR-1248, bta-miR-1434-3p, bta-miR-708, bta-miR-677, bta-miR-150,bta-miR-2484, and bta-miR-2332) formed ceRNA regulatory networks of lncRNAs-miRNAs-mRNAs (nodes with red edge in Figure 4).

### Validation of DEGs, DEmiRNAs, and DElncRNAs by RT-qPCR

**Figure 5:**
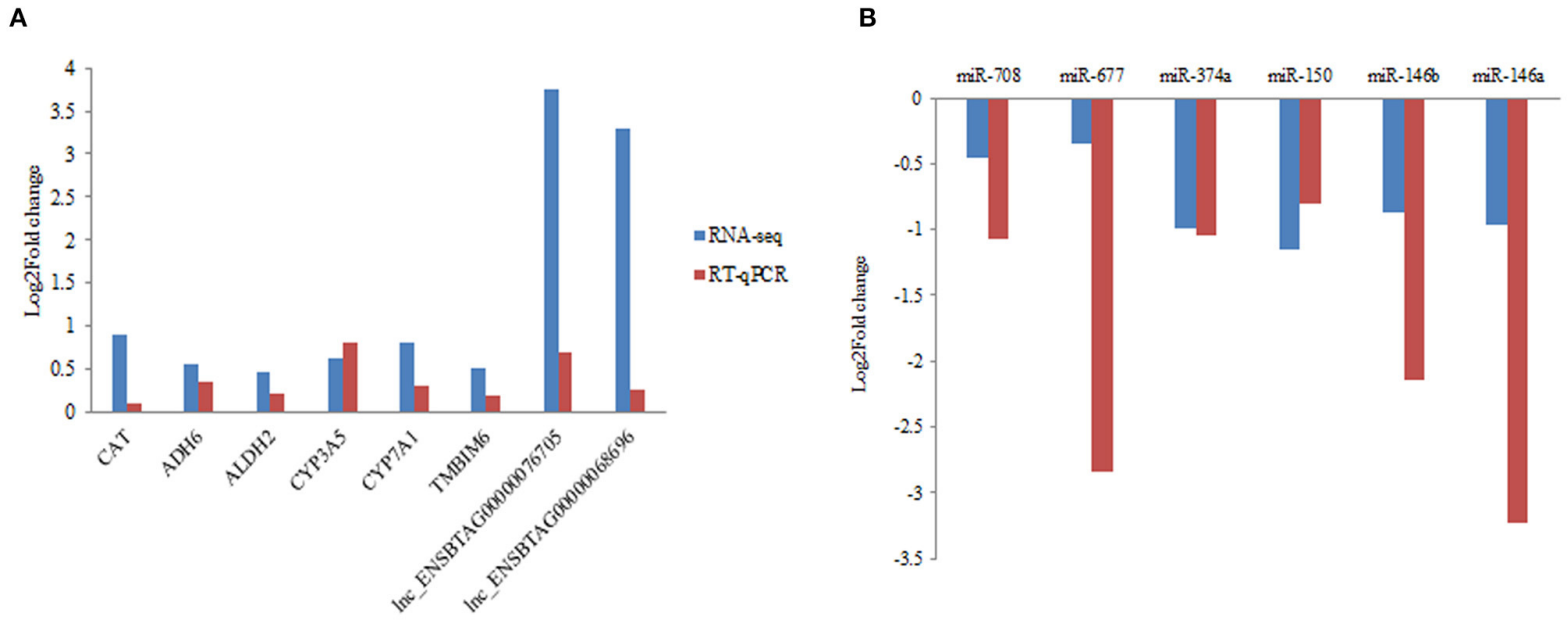
Validation of differentially expressed mRNA, miRNA, and lncRNA by RT-qPCR in liver samples. **(A)** The quantification of mRNA and lncRNA in liver from grass-fed vs. grain-fed groups. **(B)** The quantification of miRNA in liver from grass-fed vs. grain-fed group.

In the present study, RT-qPCR analysis was performed in six DEGs, six DEmiRNA with random selection, and two DElncRNAs. Primers were designed for RT-qPCR analysis (Supplementary Table 9). We confirmed the expression consistency between the RT-qPCR results and RNA-seq data from the grass-fed and grain-fed group (Figure 5).

### Carbohydrate, Cholesterol, Bile Acid, and Other Metabolites Changes Related to Energy Metabolism in Blood From Metabolomics Analysis

Metabolomics analysis was performed by GC/MS and UPLC/MS/MS. We found the glucose, pyruvate, and lactate concentrations in the carbohydrate metabolism pathway were significantly lower in the grass-fed group than that of the grain-fed group (*P* < 0.05) (Table 1). The related metabolites to tricarboxylic acid cycle (TCA) like alpha-ketoglutarate, succinylcarnitine and succinate, and oxidative phosphorylation like pyrophosphate were also significantly lower in the grass-fed group than that of the grain-fed group (*P* < 0.05) (Table 1). However, fructose and the mixed isobar were significantly higher in the grass-fed group than that of the grain-fed group (*P* < 0.05) (Table 1).

**Table 1:** Differences between carbohydrate and other energy metabolites in blood between grass-fed and grain-fed beef cattle.

**Biochemical name**	**Mean grass**	**Mean grain**	Fold change^*^	***p*-value**	***q*-value**
1,5-anhydroglucitol (1,5-AG)	0.7129	1.3391	0.53	0.0011	0.0012
glucose	0.7449	1.2976	0.57	<0.0001	0.0001
glucose-6-phosphate (G6P)	1.9050	0.7356	2.59	0.4665	0.1536
Isobar: fructose 1,6-diphosphate, glucose 1,6-diphosphate, myo-inositol 1,4 or 1,3-diphosphate	1.7824	0.6545	2.72	<0.0001	<0.0001
dihydroxyacetone phosphate (DHAP)	1.2024	1.0187	1.18	0.5649	0.1761
3-phosphoglycerate	0.8394	1.0488	0.80	0.1736	0.0673
Pyruvate	0.8469	1.9353	0.44	0.0298	0.0154
lactate	0.7172	1.0929	0.66	0.0033	0.0029
glycerate	0.9850	1.0354	0.95	0.4982	0.1610
Isobar: pentulose 5-phosphates	1.4289	1.0344	1.38	0.6027	0.1852
Ribulose	0.7385	0.8698	0.85	0.4468	0.1494
ribose	0.6935	1.4983	0.46	0.0037	0.0031
xylonate	1.2002	0.8599	1.40	0.1088	0.0458
xylose	0.9143	1.0736	0.85	0.1027	0.0437
xylitol	0.9268	1.1301	0.82	0.0291	0.0152
threitol	0.9706	1.0778	0.90	0.3613	0.1271
arabitol	0.7460	1.1139	0.67	0.0760	0.0341
fructose	1.0524	0.8536	1.23	0.0937	0.0408
sorbitol	0.8577	1.2186	0.70	0.0226	0.0124
mannose	0.8744	1.1021	0.79	0.0100	0.0067
erythronate	0.9914	0.9654	1.03	0.6685	0.2005
citrate	1.1010	0.9630	1.14	0.3635	0.1273
alpha-ketoglutarate	0.5824	1.4399	0.40	0.0001	0.0001
succinylcarnitine	0.8813	1.1859	0.74	0.0101	0.0067
succinate	0.7529	1.1785	0.64	0.0021	0.0020
fumarate	1.1280	0.8967	1.26	0.0538	0.0256
malate	0.9892	0.9472	1.04	0.4962	0.1610
acetylphosphate	0.9930	1.0004	0.99	0.8639	0.2402
pyrophosphate (PPi)	0.8382	1.3170	0.64	0.0218	0.0121
phosphate	0.9376	1.0350	0.91	0.0652	0.0304

* *The relative change of grass-fed to the grain-fed group from the mass spectrometry intensities measured in equivalent volume blood*.

The concentrations of sterol, primary and second bile acid metabolites in blood from grass-fed and grain-fed groups were shown in Table 2. The concentrations of cholesterol, beta-sitosterol, glycocholate and glycochenodeoxycholate (primary bile acid), and 7-ketodeoxycholate (second bile acid) in blood from the grass-fed group were significantly higher than that of the grain-fed group (*P* < 0.05).

**Table 2:** Changes of cholesterol and bile acid concentrations in blood from grass-fed and grain-fed beef cattle.

**Pathway**	**Biochemical Name**	**Mean Grass**	**Mean Grain**	**Fold Change**	***p*-value**	***q*-value**
Sterol	Cholesterol	1.0842	0.9563	1.13	0.0397	0.0198
	7-beta-hydroxycholesterol	1.0863	1.0956	0.99	0.8555	0.2398
	7-alpha-hydroxy-3-oxo-4-cholestenoate (7-Hoca)	0.9053	1.1463	0.79	0.0149	0.0091
	Cholestanol	0.9675	1.0234	0.95	0.6252	0.1901
	Beta-sitosterol	1.2467	0.6750	1.85	0.0010	0.0011
	Campesterol	1.1258	0.9700	1.16	0.1278	0.0528
Primary bile acid metabolism	Cholate	1.8244	0.8086	2.26	0.1001	0.0428
	Glycocholate	2.5883	0.8102	3.19	0.0053	0.0041
	Chenodeoxycholate	1.0063	0.9948	1.01	0.6748	0.2010
	Glycochenodeoxycholate	2.1267	0.7008	3.03	0.0087	0.0061
Secondary bile acid metabolism	Deoxycholate	0.9724	1.2939	0.75	0.6229	0.1901
	Glycodeoxycholate	1.2744	1.2473	1.02	0.8550	0.2398
	Taurolithocholate	1.0668	1.4461	0.74	0.2501	0.0924
	7-ketodeoxycholate	1.5185	0.4695	3.23	0.0204	0.0117

## Discussion

The liver, as a vital organ, involved in a series of metabolic and homeostatic functions (Berg et al., 2002), removal of waste products and detoxification (Moubarak and Rosenkrans, 2000), bile acid synthesis (Vessey et al., 1977), and hormone secretion (Rao et al., 1979). Our studies indicated that many mRNAs and ncRNAs expression in liver and metabolite levels in the blood of beef cattle are different under two feeding regimens, which suggested the complexity of metabolic regulation.

**Figure 6:**
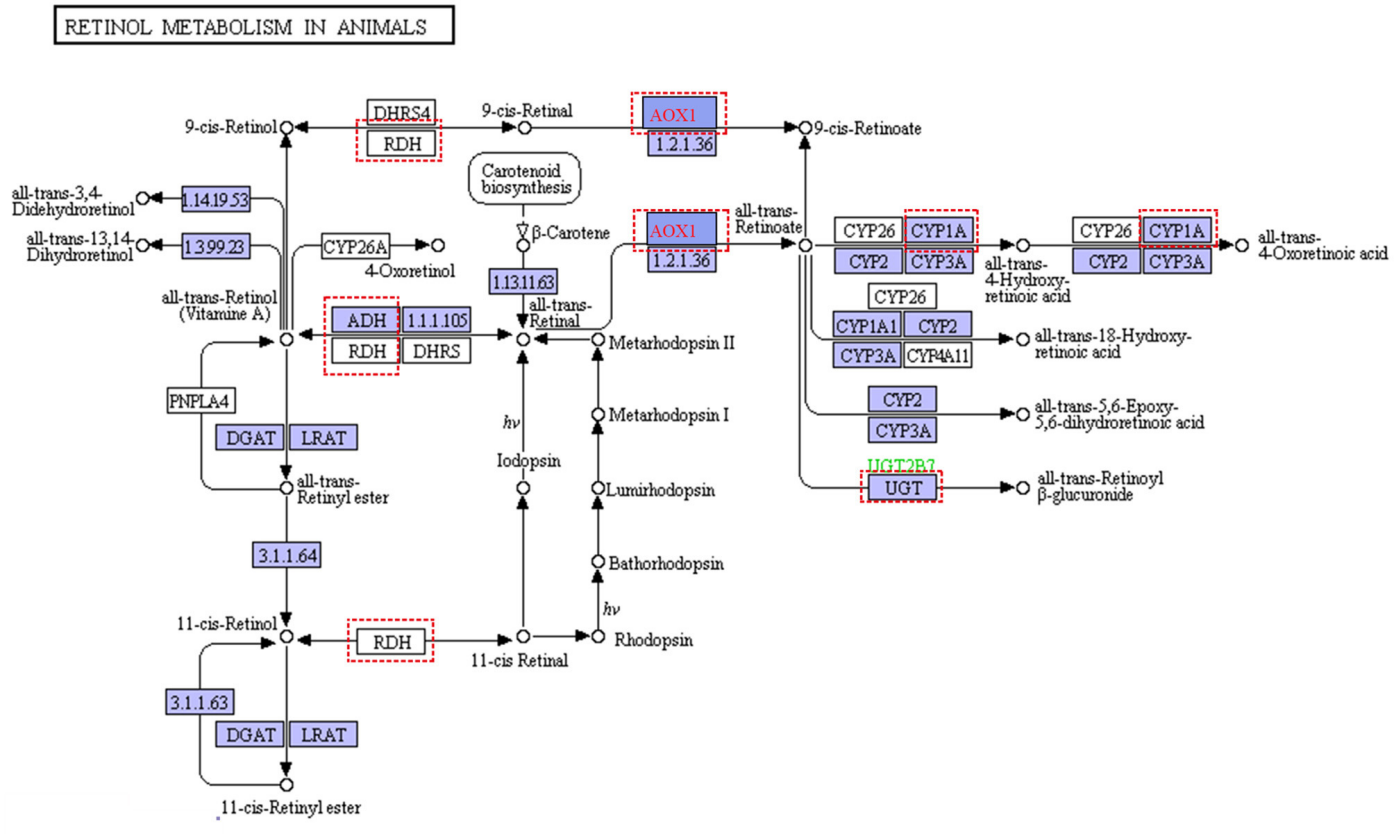
Retinol metabolism in animal (https://www.genome.jp/kegg-bin/show_pathway?ec00830+1.2.3.1). Red dashed represented differential expression genes in liver from grass-fed cattle.

For pastures, the most limiting nutrient factor is energy sources. In our study, the diet in the grass-fed group had more structural carbohydrates, and the ratio of NFC and NDF was lower than that of the grain-fed group. Glucose is an energy carrier rarely absorbed from the small intestine, especially for ruminants feeding high roughage (like grass-fed) compared with that of feeding high grain diet (like grain-fed) (McAllan and Smith, 1974). According to the metabolomics analysis results, blood glucose, pyruvate, and lactate in the grass-fed group were lower than that of the grain-fed group (Table 1). Moreover, the concentrates of metabolites from TCA and pentose pathways were also low in the grass-fed group. All these indicated that there was a demand trend of glucose for homeostasis in the grass-fed group. Our data supported the previous study that the highly expressed proteins in the low feed efficiency group were enriched glycolysis/gluconeogenesis and fatty acids degradation pathway (Fonseca et al., 2019). In our study, glycolysis, gluconeogenesis, and fatty acids degradation genes included aldolase, fructose-bisphosphate B (*ALDOB*), phosphoenolpyruvate carboxykinase 2 (*PCK2*), fructose-1,6-bisphosphatase 1 (*FBP1*), alcohol dehydrogenase 4 (*ADH4*), *ADH6*, and acetaldehyde dehydrogenase 2 *(ALDH2)*, which were up-regulated in the grass-fed group (Figure 1 and Supplementary Tables 3, 4). Aldolase B is encoded by the *ALDOB* gene, a key enzyme for fructose metabolism, and preferentially expressed in the liver. It catalyzes the specific and reversible cleavage of fructose-1,6-bisphosphate and fructose-1-phosphate into dihydroxyacetone phosphate and d-glyceraldehyde-3-phosphate, or d-glyceraldehyde for gluconeogenesis and glycolysis (Devuyst and Igarashi, 2018). Fructose-1,6-bisphosphatase 1 encoded by the *FBP1* gene catalyzes the hydrolysis of fructose 1,6-bisphosphate to fructose 6-phosphate, acting as a rate-limiting enzyme in gluconeogenesis (Granner and Pilkis, 1990). Isozymes M of phosphoenolpyruvate carboxykinase is encoded by the *PCK2* gene, which catalyzes oxaloacetate conversion to phosphoenolpyruvate, the rate-limiting step in the metabolic pathway that produces glucose from lactate and other precursors derived from the citric acid cycle (Beale et al., 2007). It indicated that grass-fed cattle needed to mobilize the genes in the liver related to glycolysis/gluconeogenesis, fatty acids degradation, and amino acid metabolism pathways to meet the energy demand. In the KEGG pathways, seven genes were enriched to retinol metabolism, including *ADH4, ADH6*, aldehyde oxidase 1 (*AOX1*), cytochrome P450 family one subfamily A member 2 (*CYP1A2*), hydroxysteroid (17-beta) dehydrogenase 6 (*HSD17B6*), retinol dehydrogenase 16 (*RDH16*), UDP glucuronosyltransferase family two-member B15 (*UGT2B15*) (Figure 6 and Supplementary Table 4). Beef cattle fed high forage rations have more yellow carcass fat than that of concentrate-fed counterparts (Daley et al., 2010), caused by carotenoids from forages. Carotenes (mainly β-carotene) are precursors of retinol (Vitamin A), which is essential for healthy vision, bone growth, reproduction, cell division, and cell differentiation (Scott et al., 1994). Diets based on grass can elevate precursors for Vitamin A, so beef from grass-fed steers is rich in vitamin A (Descalzo et al., 2005), which are healthy for people.

Besides, we found cytochrome P450 family 7 subfamily A member 1 (*CYP7A1*), *DHCR24*, and *SC5D* related to steroid biosynthesis (Supplementary Tables 3, 4) in the liver from the grass-fed group attended in cholesterol and bile acid synthesis. No matter the Bloch pathway or Kandutsch-Russell pathway, both DHCR24 and SC5D are involved (Bae and Paik, 1997). SC5D, encoded by the *SC5D* gene, catalyzes the conversion of lathosterol (Kandutsch-Russell pathway) or 24-dehydrolathosterol (Bloch pathway) into 7-dehydrocholesterol or 7-dehydrodesmosterol, which is the precursor for the synthesis of cholesterol. DHCR24, encoded by the *DHCR24* gene, is the final enzyme of the cholesterol biosynthetic Bloch pathway, and in Kandutsch-Russell pathway catalyzes the conversion of lanosterol to 24,25 dihydro lanosterol (Bae and Paik, 1997). Since cholesterol synthesis is an energetically expensive process, cooperativity would ensure that critical genes must be strongly activated to commit to cholesterol synthesis (Zerenturk et al., 2012). Impaired *SC5D* or *DHCR24* activity leads to a deficiency of cholesterol (Jiang et al., 2010; Muse et al., 2018). In this study, according to the results from the metabolomic analysis, cholesterol concentration in blood was higher for the grass-fed group than that of the grain-fed group (Table 2). Previous studies reported that beef's cholesterol content from grass/forage-fed was lower than that of grain-finished cattle (Rule et al., 2002). Steers with low feed efficiency had increased bloodstream pools of cholesterol (Montanholi et al., 2017). The concentration of cholesterol is dynamic in the liver and blood. Besides, bile acid synthesis is also recognized as a primary output pathway of cholesterol from the body (Liepa et al., 1978). This study found that *CYP7A1* encoding cholesterol 7-hydroxylase was up-regulated in the liver from the grass-fed group (Supplementary Table 4). CYP7A1 is a rate-limiting enzyme of the classic pathway of bile acid synthesis (Chiang and Ferrell, 2018). It catalyzes cholesterol to form primary bile acid: cholic (CA) and chenodeoxycholic acid (CDCA), and their conjugates Tauro(glycol)cholic acid (T(G)CA) and Tauro(glycol) chenodeoxycholic acid (T(G)CDCA), which are actively transported into bile and become part of the circulating bile acid pool. In the small intestine, T(G)CA and T(G)CDCA are converted to secondary bile acids: deoxycholic acid (DCA) and Lithocholic acid (LCA), respectively (Chiang, 2013). The classic pathway of bile acid is predominant for ruminants (Sheriha et al., 1968). From our metabolomic results, the contents of GCA and GCDCA (belonged to primary bile acid) in blood from the grass-fed group were significantly higher than that of the grain-fed group (Table 2). Still, the concentration of secondary bile acids and conjugates (DCA and GDCA) showed no difference in blood between the two groups. Previous reports, both in ruminant and human studies, showed that diet composition could affect the bile acid types (Sheriha et al., 1968; Madden, 2003). When a high fiber diet is consumed, there is a greater excretion of bile acids in feces, thus less can reach the liver for re-secretion. Reversely, for a less-fiber diet, because of dehydroxylation transited to DCA slowly in the colon, the secondary bile acid is reabsorbed and inhibits the production of primary bile acid (Sheriha et al., 1968; Madden, 2003). Recently, bile acids have been discovered as regulatory molecules. Enterohepatic circulation of bile acids plays a central role in the regulation of bile acids synthesis, fatty acid, lipid, and lipoprotein synthesis, as well as glucose metabolism in the liver (Kullak-Ublick et al., 2004). Besides, vitamin A also affected bile acid synthesis by regulating *CYP7A1* expression (Schmidt et al., 2010). Meanwhile, bile acids can promote the intestinal absorption of lipid-soluble vitamins including vitamin A. Between vitamin A metabolism and bile acid synthesis, there is a negative feedback regulatory relationship.

Like diet, nutrients, environment, and management, many factors can alter gene expression by epigenetic modulations (Tarallo et al., 2014; Law and Holland, 2018). Though the number of samples was relatively small, our data provided initial analysis on epigenetic regulation mechanism. The results still showed some valuable information. Noncoding RNAs like miRNAs and lncRNAs were one of the modification components of gene expression regulation. In the present study, we identified 76 DEmiRNAs (Figure 2, Supplementary Table 6) and two DElncRNAs in the grass-fed vs. grain-fed group. In the metabolic processes and pathways networks, we found many genes were regulated by one or many miRNAs and lncRNAs (Figure 4). *CYP7A1* was regulated by three miRNAs (bta-miR-2484, bta-miR-27a-3p, and bta-miR-194) and one lncRNA in the grass-fed group. RNAs also influence each other's levels by competing for a limited miRNA pool (Salmena et al., 2011). Based on the interaction network, we found two lncRNAs and eight genes might act as ceRNA to bind miRNA (Figure 4), which affected gene expression.

## Conclusions

Our results indicated grass-fed induced the gene expression in glycolysis/gluconeogenesis, fatty acids degradation, and amino acid metabolism pathway in the liver to meet energy demand and maintain glucose homeostatic, and consequently improve beef quality. These genes were related to epigenetic regulation, which may offer new perspectives on different feeding regimens inducing metabolic regulation.

## Data Availability Statement

The datasets presented in this study can be found in online repositories. The names of the repository/repositories and accession number(s) can be found here: https://www.ncbi.nlm.nih.gov/, GSE145376; https://www.ncbi.nlm.nih.gov/, GSE145377.

## Ethics Statement

The animal study was reviewed and approved by the Institute of Animal Care and Use Committee at the University of Maryland. Written informed consent was obtained from the owners for the participation of their animals in this study.

## Author Contributions

JS designed the experiments. CJ and JS analyzed the data and wrote the manuscript. LL, WC, and YH gave some help when analyzing data. CJ, JL, and YB performed the main experimental results. All authors read and approved the final manuscript.

## Conflict of Interest

The authors declare that the research was conducted in the absence of any commercial or financial relationships that could be construed as a potential conflict of interest.

## Supplementary Material

Supplementary Figure 1 Top 10 significantly enriched function from differential interaction genes with two lncRNAs. Biological process **(A)**, cellular component **(B)**, molecular function **(C)**, and KEGG pathways **(D)** for grass-fed vs. grain-fed group.

## Abbreviations

DEGs: differentially expressed genes
DEmiRNA: differentially expressed miRNA
ncRNAs: noncoding RNAs
lncRNA: long noncoding RNA
DElncRNA: differentially expressed lncRNA
ceRNA: competing endogenous RNAs
NFC: non-fibrous carbohydrates
NDF: Neutral Detergent Fiber
MREs: microRNA response elements
GO: gene ontology
BP: biological processes
CC: cellular component
MF: molecular function
*ADH6*: alcohol dehydrogenase 6
*AOX1*: aldehyde oxidase 1
*CYP7A1*: cytochrome P450 family 7 subfamily A polypeptide 1
*DHCR24*: 24-dehydrocholesterol reductase
*DPYS*: dihydropyrimidinase
*FBP1*: fructose-bisphosphatase 1
*GATM*: glycine amidinotransferase
*HSD17B6*: hydroxysteroid (17-beta) dehydrogenase 6
*KMO*: kynurenine 3-monooxygenase
*SC5D*: sterol-C5-desaturase
*ALDOB*, aldolase: fructose-bisphosphate B
TCA: tricarboxylic acid cycle
*FBP1*, Fructose-1: 6-bisphosphatase 1
*PCK2*: phosphoenolpyruvate carboxykinase 2
*CYP1A2*: cytochrome P450 family 1 subfamily A member 2
*RDH16*: retinol dehydrogenase 16
*UGT2B15*: UDP glucuronosyltransferase family 2 member B15
*SULT1B1*: sulfotransferase family 1B member 1
*CCL3*: C-C Motif Chemokine Ligand 3
*RBPJ*: Recombination Signal Binding Protein For Immunoglobulin Kappa J Region
*TEFM*: mitochondrial transcription elongation factor
CA: cholic acid
CDCA, chenodeoxycholic acid, T(G)CA: tauro(glyco)cholic acid
T(G)CDCA: tauro(glyco) chenodeoxycholic acid
DCA: deoxycholic acid
LCA: Lithocholic acid.
