# Supplementary figures and images for: Metabolic Regulations by lncRNA, miRNA, and ceRNA Under Grass-Fed and Grain-Fed Regimens in Angus Beef Cattle
Source: Front Genet. 2021 Mar 4;12:579393. doi: 10.3389/fgene.2021.579393 (PMC7969984; doi:10.3389/fgene.2021.579393)

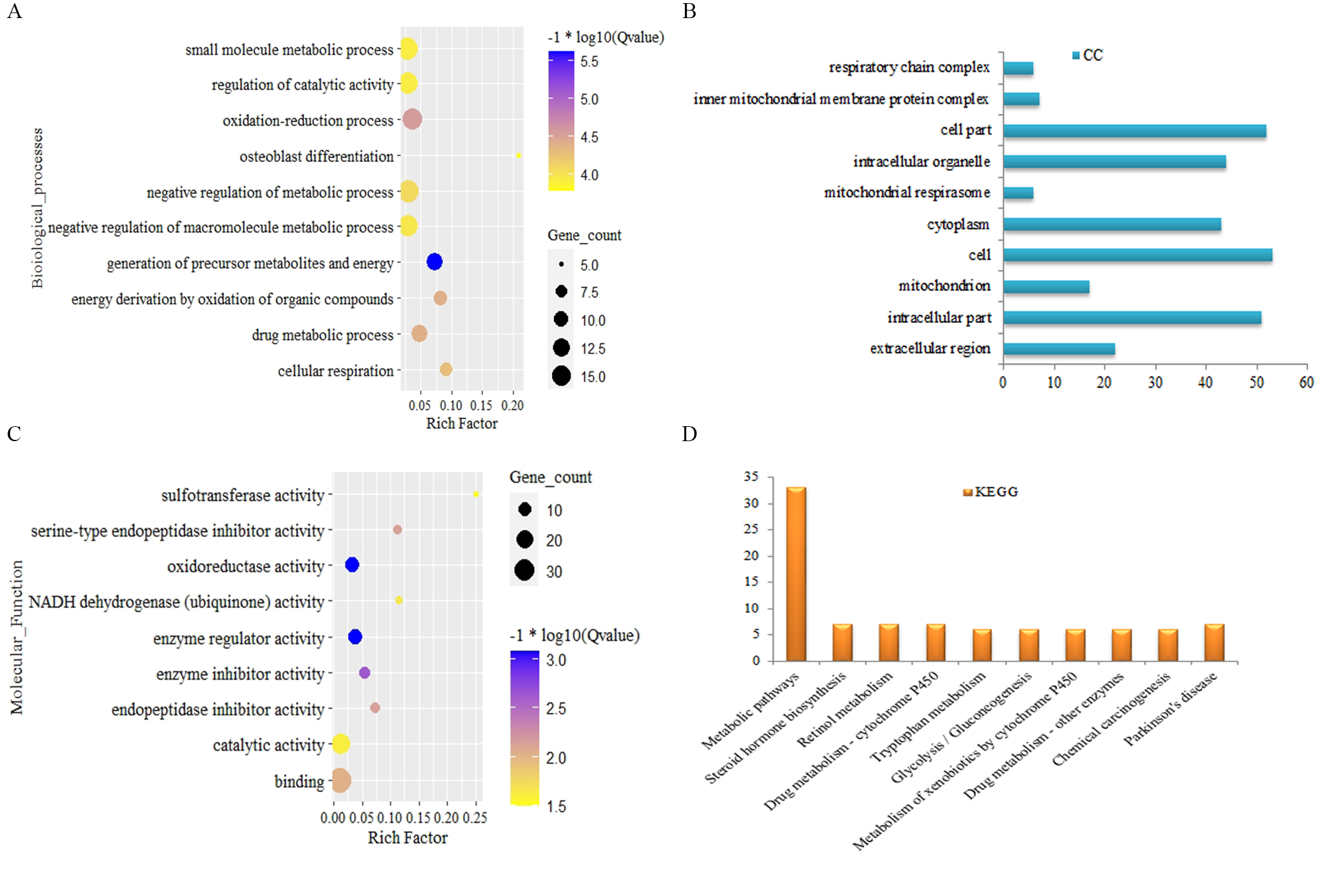

Supplement: Supplementary Figure 1 — Top 10 significantly enriched function from differential interaction genes with two lncRNAs. Biological process (A), cellular component (B), molecular function (C), and KEGG pathways (D) for grass-fed vs. grain-fed group. [file Data_Sheet_1.ZIP › Figure S1.tif]
